# 3D tumor angiogenesis models: recent advances and challenges

**DOI:** 10.1007/s00432-021-03814-0

**Published:** 2021-10-06

**Authors:** Sharath M. Bhat, Vaishnavi A. Badiger, Sampara Vasishta, Juhi Chakraborty, Seetharam Prasad, Sourabh Ghosh, Manjunath B. Joshi

**Affiliations:** 1 Department of Ageing Research, Manipal School of Life Sciences, Manipal Academy of Higher Education, Manipal, 576104 India; 2 Regenerative Engineering Laboratory, Department of Textile and Fibre Engineering, Indian Institute of Technology, Delhi, 110016 India; 3 Department of Surgery, Kasturba Medical College, Manipal Academy of Higher Education, Manipal, 576104 India

**Keywords:** 3D model systems, Organoid models, Organ on chip, Tumor angiogenesis

## Abstract

The development of blood vessels, referred to as angiogenesis, is an intricate process regulated spatially and temporally through a delicate balance between the qualitative and quantitative expression of pro and anti-angiogenic molecules. As angiogenesis is a prerequisite for solid tumors to grow and metastasize, a variety of tumor angiogenesis models have been formulated to better understand the underlying mechanisms and associated clinical applications. Studies have demonstrated independent mechanisms inducing angiogenesis in tumors such as (a) HIF-1/VEGF mediated paracrine interactions between a cancer cell and endothelial cells, (b) recruitment of progenitor endothelial cells, and (c) vasculogenic mimicry. Moreover, single-cell sequencing technologies have indicated endothelial cell heterogeneity among organ systems including tumor tissues. However, existing angiogenesis models often rely upon normal endothelial cells which significantly differ from tumor endothelial cells exhibiting distinct (epi)genetic and metabolic signatures. Besides, the existence of intra-individual variations necessitates the development of improved tumor vascular model systems for personalized medicine. In the present review, we summarize recent advancements of 3D tumor vascular model systems which include (a) tissue engineering-based tumor models; (b) vascular organoid models, and (c) organ-on-chips and their importance in replicating the tumor angiogenesis along with the associated challenges to design improved models.

## Introduction

Angiogenesis, the process of formation of new blood vessels from pre-existing vasculature plays a crucial role in health and disease. Otherwise referred to as neovascularization, the biological process of angiogenesis is considered important for normal physiological growth, tissue regeneration, and wound healing (Otrock et al. 2007). The rate of angiogenesis in different tissues is precisely modulated by the interplay between the pro- and anti-angiogenic molecules (Carmeliet and Jain 2000). Dysregulation in angiogenesis overt to a variety of pathological conditions (Ana Moraga 2017) and accordingly, the disparity in the expression of angiogenic regulators leads to aberrant angiogenesis which subsequently results in the onset of pathological conditions such as delayed wound healing, ischemic heart diseases, stroke, diabetic vasculopathy, solid tumors, rheumatoid arthritis and other inflammatory disorders (Carmeliet 2005). Both insufficient and excessive angiogenesis results into distinct pathological conditions. Therefore, cell/tissue culture models precisely mimicking patho(physiological) angiogenesis are of significant clinical importance enabling to understand the underlying mechanisms and to examine efficiency of therapeutic angiogenic modulators.

Recent developments in tumor biology have shown diverse cells (including cancer cells, cancer-associated- adipocytes, fibroblasts, macrophages, neutrophils, and endothelial cells) alter the secretome, proteome, metabolome, and degradome of the tumor niche, thereby remodeling the morphology and functionality of the tumor vasculature (Riabov et al. 2014; Hida et al. 2016; Masucci et al. 2019; Wang et al. 2019). Tumor vasculature is characterized by the presence of irregularly shaped discontinuous endothelium and abnormal basement membrane deposition producing “leaky” and torturous blood vessels with compromised circulatory function (Siemann 2011). These leaky blood vessels irrigating the tumors not only assist abnormal growth and development but also facilitate metastasis and resistance against anti-cancer agents. Nagy et al. reported the presence of six morphologically and functionally distinct tumor vessel subtypes including mother vessels, capillaries, glomeruloid microvascular proliferations, vascular malformations, feeder arteries, and draining veins; of which two of the vessel forms were capable of surviving in the absence of exogenous VEGF-A (Nagy et al. 2009). Employing a breast cancer model, Chen et al. demonstrated that the enhanced expression of VEGF and the recruitment of endothelial cells led to the changes in the architecture and polarity loss during the breast cell transformation. Interestingly the expression of VEGF was reduced by reverting the malignant phenotype upon reexpression of HoxD10 (Chen et al. 2009). This clearly demonstrated the significance of tissue architecture and polarity in moderating the angiogenic switch in the course of malignant transformation.

Many studies have reported the role of tumor vasculature in restraining the efficacy of the anti-cancer drug by limiting their reach in the tumors (Dey et al. 2015). Local hypoxia caused by insufficient oxygenation due to vessel leakiness resulted in the formation of an immuno-attractive microenvironment leading to the recruitment of immunosuppressive immune cells (Schaaf et al. 2018). These studies indicated that tumors thrive on dysregulated angiogenesis for (a) growth, development, and metastasis, (b) recruitment of diverse cell types to support their survival and (c) anti-cancer drug resistance. Drugs with the ability to normalize the tumor vasculature hinder these functions and hence serve beneficial in developing treatment strategies (Carmeliet 2005).

The development of experimental model systems with the ability to resemble various patho(physiological) angiogenic tumor microenvironments is a prerequisite for a better understanding of mechanisms governing the vascular intricacies. The most extensively used model system were the 2-dimensional (2D) primary cell culture models of human umbilical vein endothelial cells (HUVEC), and dermal microvascular endothelial cells (HDMEC), which contributed to most of our knowledge on tumor endothelial cell biology. Further, 3-dimensional (3D) cultures were also developed which enabled co-culturing endothelial cells along with other cell types such as pericytes, epithelial cells, fibroblasts, or tumor cells, which increased our knowledge on paracrine interactions of the endothelial cells. Simultaneously, ex vivo models such as Matrigel plug assays and chicken chorioallantoic membranes were established, to understand underlying cellular mechanisms and to screen various angiogenic drugs in pre-clinical experiments (Stryker et al. 2019). However, all these aforesaid models were unable to completely recapitulate the tumor microenvironment due to a lack of 3D cellular organization and specific cellular orientations. In recent years, advancement in cell and tissue engineering has led to the development of organoid models, which are constructed by depositing heterogeneous cell types in a pre-defined matrix of biomaterials (Gupta et al., 2018). These organoid models often employ patient-derived induced pluripotent stem cells (iPSCs), which enables the formulation of personalized drug profiles to develop precision medicine protocols. On the other hand, deposition of biomaterials in a pre-defined matrix determines the precise structure of the organoid facilitating functional replacement of the patho(physiological) tissue/organ. Further, the application of 3D bioprinting strategies enables us to precisely control the orientation of cell as well as ECM, homotypic/heterotypic 3D cellular interactions to closely mimic their parental organ. Accordingly, vascular organoid/bioprinted models might facilitate a better understanding of tumor vascular biology and associated functions.

In the present review, we have summarized (a) existing models of tumor angiogenesis along with their advantages and disadvantages, (b) recent advances in the formulation of 3D vascular model systems, and (c) challenges in constructing improved 3D tumor vascular models. Further, we attempted to elucidate how these vascular organoids can be explored to study underlying cellular and molecular mechanisms in disorganized and functionally aberrant tumor vasculature. Interestingly, recent developments in genomics have revealed significant endothelial heterogeneity and intra-individual variations. Hence, in this review, we have also proposed the need for developing tissue-specific and personalized vascular models in the tumor perspective manner.

**Fig.1:**
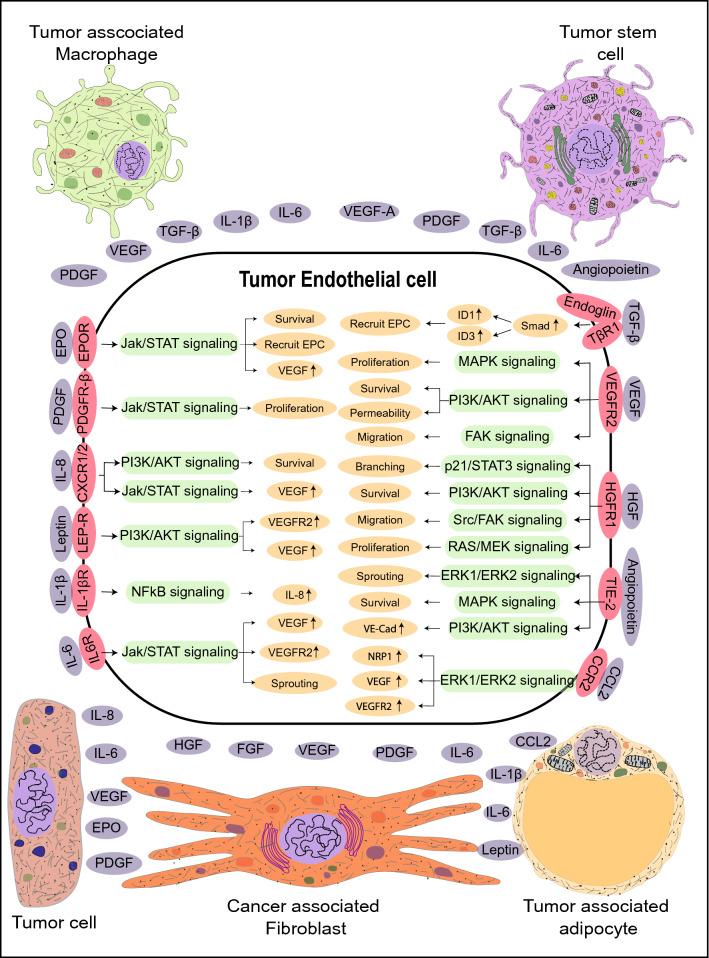
Tumor angiogenic regulators differ from that of physiological angiogenesis and other pathologies. In the tumor microenvironment, immune and non-immune cells secrete diverse angiocrine factors to induce disordered angiogenesis

**Fig. 2:**
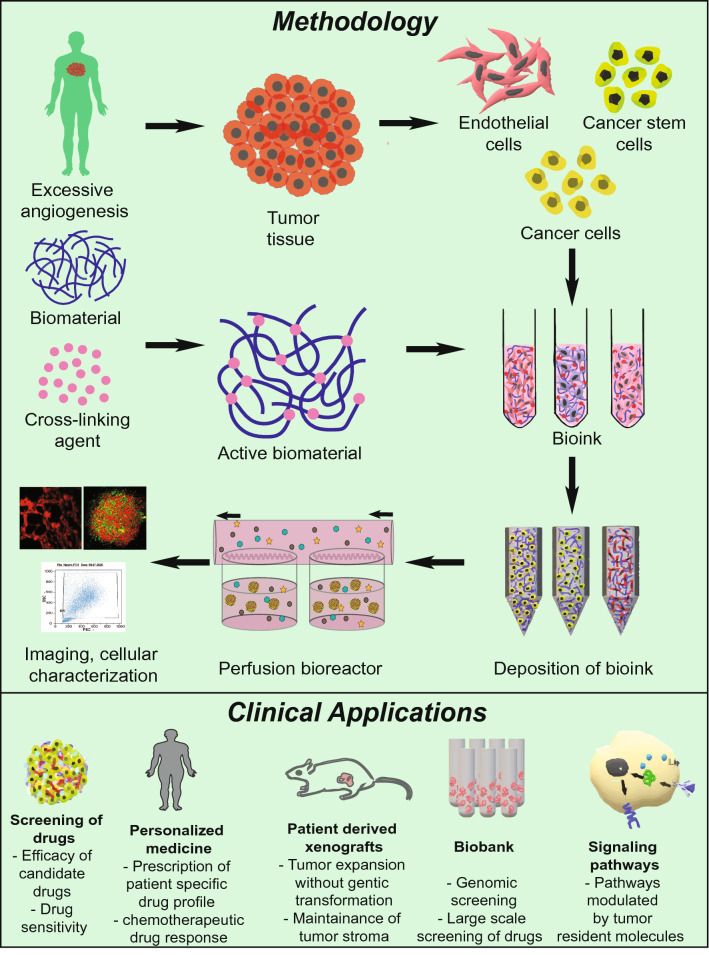
Work flow to formulate 3D tumor vascular model systems and their clinical applications

1. Angiogenic modulators vary in tumors compared to other tissues: the need for developing tumor-specific vascular models
  To fulfill the nutritional demands of the tissues, including solid tumors a functional vasculature is essential. In normal physiology, neoangiogenesis is involved in the development of the embryo and wound healing. Pathological angiogenesis of either excessive (tumors, diabetic retinopathy) or insufficient (ischemic diseases) vessel growth is a consequence surrounding aberrated secretome. Excessive dysregulated angiogenesis in solid tumors is modulated by the expression of members of vascular endothelial growth factor (VEGF), fibroblast growth factor (FGF), platelet-derived growth factor-A (PDGF-A), hepatocyte growth factor (HGF), transforming growth factor-β (TGF-β) and interleukin family of cytokines (Aharonov et al. 1993; Viallard and Larrivée 2017) (Fig. 1). Recently we showed that interleukin-6 (IL-6) induced DNA hypomethylation of VEGFR2 promoter in endothelial cells isolated from clinically characterized human breast tumors and facilitated disordered angiogenesis in 3D spheroid models (Hegde et al. 2020). Elevated expression of angiocrine molecules in the tumor niche is often coupled with the downregulation of anti-angiogenic molecules such as endostatin, matrix metalloproteinases (MMP) inhibitors, tissue inhibitors of metalloproteinases (TIMPs), thrombospondin-1 and interferon-alpha (IFN-α) (Ren et al. 2006; Wong et al. 2009). The hypoxic microenvironment of the tumor induces the expression of Hypoxia-inducible factor (HIF)-1α, which leads to transcriptional upregulation of VEGF-A in the cancer cells (Carmeliet and Jain 2000). Subsequent, binding of VEGF-A to its cognate receptor VEGFR2 on the endothelial cells (EC) of the neighboring blood vessels induces the expression of genes involved in EC proliferation and migration (Stacker and Achen 2013). Elevated levels of VEGF in the tumor microenvironment downregulates PDGF in ECs which results in its loss of interaction with pericytes causing “leaky” blood vessels, a hallmark of cancers (Viallard and Larrivée 2017). Along with sprouting and intussusceptive types of angiogenesis, vasculogenic mimicry (VM) and vasculogenesis enable the fulfillment of the oxygen demand and nutrient requirements of the tumor. The hypoxic microenvironment of the tumor promotes the malignant cells to dedifferentiate and express endothelial cell markers such as von Willebrand factor (vWf), vascular endothelial-cadherin, CD31, laminin 5 γ2-chain, and EphA2 resulting in the formation of ‘endothelial cell-like cells’ (Dunleavey and Dudley 2012). These cells form tubular vessel-like structures mimicking vasculature, fulfilling the nutritional needs of the tumor. Increased expression of VEGF-A and SDF-1 locally in the tumor microenvironment recruits circulating EPCs to the tumors and undergo the process of neovascularization (Zhao et al. 2016).
  Pathological conditions including atherosclerosis, stroke, and delayed wound healing occur due to a shift in the molecular equilibrium of pro-angiogenic and anti-angiogenic factors leading to insufficient angiogenesis. Conditions such as intra-arterial occlusion of the coronary artery, leading to loss of cardiac function (Khurana et al. 2005) and occlusion of the cerebral vasculature, leading to stroke can be fatal. Failure to re-establish a functional vasculature due to inadequate angiogenesis results in ischemia, causing permanent damage to the affected tissue, compromising its functionality (Chertok et al. 2019). Excessive angiogenesis, a characteristic feature of disorders such as diabetic retinopathy, rheumatoid arthritis, and solid tumors is also caused by dysregulation of the angiogenic signaling pathways. For example, both transcriptional and translational upregulation of VEGF-A and vascular endothelial growth factor receptor-2 (VEGFR2) in diabetic vasculopathy, such as proliferative retinopathy, results in excessive angiogenesis. Significant downregulation of PDGF-A, stromal cell-derived growth factor-1 alpha (SDF-1α) and other angiogenic cytokines results in the formation of disorganized blood vessels leading to vascular dysfunction in the context of diabetes (Kolluru et al. 2012; Okonkwo and DiPietro 2017). Earlier studies in our lab in the context of vascular insulin resistance have demonstrated IL-6 induces DNA methylation based epigenetic changes of pro-angiogenic genes in human endothelial cells upon degrading the DNA methyltransferase 1 isoform (Balakrishnan et al. 2018).
  As discussed above, angiogenic response varies spatially and temporally under various pathological and physiological conditions. Taken together, the molecular aberrations associated with angiogenesis in solid tumors differ from other diseases such as coronary heart disease, stroke, diabetic vasculopathy, and rheumatoid arthritis Hence, understanding these disease-specific signaling mechanisms governing angiogenesis will not only help us to develop novel drug targets but will also help us to formulate tissue-specific and patient-specific treatment profiles for precision medicine.
2. Existing models for tumor angiogenesis: advantages and disadvantages
  Endothelial cells, pericytes, vascular smooth muscle cells, and extracellular matrix are the key cellular components of a healthy vasculature. As endothelial cells are one of the primary components of the blood vessels, most of the in vitro model systems of angiogenesis focus on endothelial cell proliferation, migration, and tubule formation assays. Morphological and functional heterogeneity along the vascular tree has been well documented (Gerritsen 1987; Castro et al. 2018). Morphologically, continuous endothelial cells are associated with tight junctions and attached to a continuous basal membrane. This forms the innermost layer of the blood vessels including arteries, veins, and capillaries of various organs such as the brain, heart, and lungs; fenestrated endothelial cells on the other hand are present in tissues such as exocrine glands, the gastrointestinal tract where elevated trans-endothelial transport occurs. Finally the discontinuous endothelial cells are characterized by the presence of large 100–200 nm fenestrations without diaphragm and have discontinuous basal lamina which is found in liver, bone marrow, and spleen. Endothelial cells are functionally heterogeneous and play different roles in controlling hemostasis, fibrinolysis, thrombosis, antigen presentation, vasoconstriction, vasodilation, and atherogenesis (Staton et al. 2009). Though considerable heterogeneity exists in ECs, the most frequently used ECs for in vitro assays only include HUVECs and (Jaffe et al. 1973) HDMECs isolated from human umbilical veins, and foreskin tissue, respectively (Goodwin 2007).
  In vivo assays are commonly used to explore the effect of drugs on tumor angiogenesis and to validate the findings of the molecular mechanisms from in vitro and ex vivo studies. Conventionally, tumors are grown subcutaneously, orthotopically, or as xenografts in immunodeficient recipients and are examined for desired effects. For example, Michael et al*.,* implanted fragments of Brown-Pearce epithelioma and V2 carcinoma into rabbit corneal stroma and demonstrated the process of neovascularization in an avascular site (Gimbrone Jr. et al. 1974). Transgenic nude mice ubiquitously expressing green fluorescent protein (GFP), were transplanted with red fluorescent protein (RFP) expressing cancer cell lines, for better visualization of cellular interactions between the host and the tumor (Yang et al. 2004). Nude mouse strains expressing GFP under various endothelial-specific gene promoters such as Nestin regulatory protein, endothelial nitric oxide synthase (eNOS), and Tie2 have been successfully developed and are widely being used to study tumor vessel characteristics and screening anti-angiogenic drugs (Okabe et al. 1997; Staton et al. 2009). Though in vivo models compensate the shortcomings of the in vitro models such as 3D architecture, better physiological replica, and influence of diverse systemic factors, they still fail to faithfully replicate the parent tissue due to reproducibility, biokinetic parameters, intraspecific variations, and structural and functional dissimilarity between the parental and the experimental tissue.
  Ex vivo assays enable the spatial organization of various cell types and extracellular matrices along with spatial–temporal gradients of angiogenic factors. Several ex vivo assays including rat aortic ring assay, chick aortic arch assay, and mouse fetal metatarsal angiogenesis assays have been employed for anti-angiogenic drug screening and to elucidate molecular mechanisms governing tumor angiogenesis (Tufan and Satiroglu-Tufan 2005). For example, Aplin et al., used co-cultures of rat aortic rings and cancer cell lines MDA-MB-231, OVCAR, and PC3 and showed that angiogenic sprouts arise as early as 2 days from the cut edges of the explants. However, the authors also observed that the vascular spouts regressed after 8 days of culture (Aplin and Nicosia 2016). Oehler et al*.,* used a subcutaneous sponge angiogenesis assay employing adrenomedullin transfected endometrial carcinoma cells in athymic mice to demonstrate the pro-tumorigenic and pro-angiogenic activity of adrenomedullin (Oehler et al. 2002). Semba et al*.,* used dorsal air sac model to validate the anti-angiogenic activity of E7820 against subcutaneously grown human pancreatic cancer cell line KP-1 and AsPC-1, colorectal cancer cell lines WiDr, Colo320DM and LoVo, breast cancer cell line BT20, and Kidney cancer cell line RCC-1 (Semba et al. 2004). Although over the years various ex vivo assays are being explored to understand vascular functions, these models have limited clinical applications as these models are unable to contemplate the tumor heterogeneity, genetic, and epigenetic disparities between primary tumors and cell lines, intraspecific variations, spatial divergence from the parental tumor and graft rejection by the host. Altogether existing in vitro*, *in vivo*,* and ex vivo models (a) do not allow the study of paracrine interactions; (b) do not mimic in vivo cellular spatiotemporal orientation and (patho)physiological microenvironment and (c) might not be extrapolated to clinical models. The advantages and disadvantages of various existing in vivo*, *in vitro*,* and ex vivo angiogenic models are summarized in Table 1.
  Recent advances in constructing vascular 3D models include (a) Tissue-engineered vascular models which utilize terminally differentiated endothelial cells or progenitor endothelial cells; (b) organoid models using endothelial progenitor cells; and (c) organ-on-chips that employ both terminally differentiated and endothelial progenitor cells. All these models are constructed along with other heterogeneous cell types depending on the tissue/organ models.
3. Tissue-engineered tumor vascular models
  Formulation of biomimetic model systems employing suitable cells along with compatible biomaterials with precise control on biochemical and physiochemical properties have been made possible with the help of available tissue engineering techniques which have been explored for a wide variety of purposes including tissue replacement, clinical diagnosis, and *inter alia* (Fig. 2) (Chen and Liu 2016; Shirure et al. 2021). Methods to vascularize various tissue/organ types using several synthetic and naturally occurring biomaterials have already been established. For example, Lazzari et al., constructed a poly-HEMA-based 3D tumor model by co-culturing PANC-1, MRC-5, and HUVEC to synthesize vascularized tumor spheroids of pancreatic cancer cells (Lazzari et al. 2018). Bray et al. developed a bioengineered star PEG-heparin hydrogel system equipped with tunable biochemical as well as mechanical properties that demonstrated the unique feature of replicating the tumor angiogenesis using HUVEC cells as well as in vivo drug response (Bray et al. 2015). In a follow-up study, it was shown that the invasive cancer cells resulted in phenotypically aggressive tumors that resulted when peptides got embedded and started replicating the natural ECM sites for example the collagen and laminin. The resultant invasive cancer cells were superior at inducing angiogenesis in comparison to their noninvasive counterparts (Perou et al. 2000). 3D bioprinting constitutes the fabrication of a pre-defined matrix by the deposition of hydrogels containing either one cell type or a mixture of different cell types in the form of a thin filament, either by fused filament fabrication or fused deposition modeling. Bioprinting offers a paradigm shift in the field of organoid development by offering prospects for simulating the early phases of in vivo organogenesis (Chawla et al. 2018a, b). 3D bioprinting is proven beneficial in cases where exogenous administration of growth factors may lead to undesired effects. Various growth factors are covalently or non-covalently immobilized with the bioink for their delivery in a controlled spatiotemporal manner. As a result, angiogenesis is promoted for an extended time period as compared to traditional scaffold-based approaches. Moreover, bioprinting offers the possibility of controlling the alignment of cells, as well as to strategically modulate cellular signaling pathways; thus recapitulating the specific microstructure of target tissue or organ compared to standard tissue fabrication approaches (Chakraborty and Ghosh 2020).
  Bio-inks are the key elements of any bioprinting process, in which the cells are encapsulated and extruded to develop the tissue or organ of interest. Alginate is frequently used as a bioink with calcium chloride that acts as a support material as well as a cross-linking agent. For example, Ferreira et al*.,* constructed bevacizumab loaded alginate hydrogel which inhibited tumor angiogenesis by a pH-dependent controlled release of bevacizumab in CAM assay (Ferreira et al. 2017). Similarly, Fischbach et al*.,* used RGD (an integrin recognition site) bound alginate hydrogel to demonstrate enhanced secretion of IL-8 and VEGF by OSCC-3, MDA-MB-231 and glioblastoma cell line U87, whose blockage significantly reduced tumor angiogenesis and growth (Fischbach et al. 2009). Further, *Bombyx mori* silk fibroin and gelatin (SF–G) hydrogels have been used extensively in the past to develop bioinks for extrusion-based bioprinting (Chawla et al. 2018a, b). SF-G bioink has been used to encapsulate both single-cell suspensions as well as cellular organoids for the development of tissue equivalents like cartilage, bone, and skin as well as disease models (Sasmal et al. 2018). Yan et al. used HeLa cell-laden silk fibroin hydrogel implants in CAM models to show the induction of apoptosis mediated by conformational changes in the 3D structure of the hydrogel. The authors revealed that the transparent, elastic pH-responsive random coil conformation supports cell survival but later its transition to the β-sheet conformation induces apoptosis in vitro and inhibits angiogenesis and tumor growth in vivo (Yan et al. 2016). Furthermore, single cells, as well as self-assembling tissue spheroids, have also been utilized to develop vascular grafts. For example, Wartenberg et al. cultured human embryonic stem cell line H1 with tumor spheroids of human prostate cancer cell line DU-145 to demonstrate infiltration of CD31^+^ cells into the tumor spheroids suggesting the use of these confrontational cultures to evaluate anti-angiogenic drugs and to study expression patterns due to cellular interactions (Wartenberg et al. 2006). Droplet-based bioprinting methods have been utilized to develop 3D bioprinted vascular constructs with enhanced resolution. For example, Meng et al. built tumor constructs of human adenocarcinoma alveolar basal epithelial cell line A549 in gelatin methacrylate bioink to recapitulate the complexity of the tumor microenvironment. The constructs comprising tumor cells, HUVECs, and fibroblasts were treated with laser inducible capsules of signaling molecules such as EGF and VEGF to create a chemical gradient. The authors, using this 3D vascular tumor model, demonstrated tumor defining features such as metastasis, angiogenesis, intravasation and also tested immunotoxin EGF4KDEL which inhibited tumor growth (Meng et al. 2019). Moreover, a more recent advancement in the field of bioprinting called freeform reversible embedding of suspended hydrogels has been used for the development of tubular structures (both straight and branched) for instance compound bioink consisting of a mixture of alginate and decellularized porcine aortic tissue ECM based bio-blood vessels (Gao et al. 2017). DelNero et al. constructed an alginate-based 3D vascularized tumor model capable of maintaining a homogenous oxygen level. Employing these models, the authors demonstrated tumor cells derived IL-8 along with VEGF markedly upregulated the invasiveness of endothelial sprouts (DelNero et al. 2015). Thus, bioprinting combined with appropriate bioink compositions as well as suitable cells or cellular spheroids holds the potential to develop functional tumor vascular grafts for disease modeling purposes. Further, employing these tumor vascular models along with organoids of multiorgan systems to simulate paracrine and endocrine signaling might enable us to study the influence of distant organs in regulating various aspects of tumorigenesis.
  It is important to mention here that despite the fascinating prospects, a lot of optimizations for bioprinting parameters such as concentration of the hydrogel, rheology of the bioink, extrusion pressure, and speed and cellular viability limits the progress of this field. A heterogeneous mixture of isolated cells is proportionately mixed with desired biomaterials to produce bioink. This can later be bioprinted using various available techniques to formulate tumor vascular organoids demonstrating pathological conditions of excessive angiogenesis. A summary of vascularized tissue-engineered models developed in recent years using different biomaterials along with their applications in various fields of biology is summarized in Table 2.
  Nevertheless, there are many interesting but ignored aspects that could raise the level of tissue-engineered tumor vascular grafts for instance by assisting in pre-vascularization of the developed tumor grafts, mechanical stimulation of bioprinted constructs that could help control the branching/sprouting of the blood vessel. The mechanical stimulation in tumor tissue is mediated by its ECM and thus the mechanical properties of the tumor ECM largely influence the behavior of constituent cells. For instance, various studies have shown growth and maturation of tumor endothelial cells that are influenced by mechanical stress on the inner vessel wall and extravascular mechanical stress (Egginton et al. 1998; Shiu et al. 2005; Giverso and Ciarletta 2016). Moreover, studies have reported that tumor constituent cells regulate the biomechanical properties of the ECM, decreases the ECM stiffness by increasing the matrix metalloproteinase (MMPs) such as MMP2, MMP9, MMP13, and MMP14 enabling them to metastasize to distant tissues (Northcott et al. 2018; Li and Wang 2020). Further, a few studies have highlighted the prospective of electrical stimulation in promoting angiogenesis, however, most of these studies are preliminary in vitro studies, for instance, basic alignment studies under directional electrical fields of 150–400 mV/mm for different cell types like pulmonary artery fibroblasts, murine aorta SMCs and HUVECs (DC electric fields induce distinct preangiogenic responses in microvascular and macrovascular cells). The success of these preliminary studies pave way for further detailed studies combining the positive aspects of mechanical and electrical stimulation with tissue engineering in promoting vascular cell organization and microvessel alignment in vitro to construct better tumor vascularized models*.* Further*,* another least explored area is the effect of surface topography, the nano/micron-scale in vivo 3D topographical features that the ECM proteins are assembled into. Thus, scaffolds recapitulating topographical features akin to native ECM might help to modulate the orientation of the microvascular network in vitro.
4. Tumor spheroid models to understand underlying mechanisms of angiogenesis
  2D cell culture techniques do not faithfully replicate the in vivo mechanical and biochemical cellular and molecular interactions which lead to alterations in cell morphology, rate and plane of cell division, and also their physiological function. The presently available scaffolds makes it cumbersome to obtain a controlled matrix that can sustain both cellular physiological growth as well as interaction similar to that observed in vivo (Cunha et al. 2011; Jaganathan et al. 2014). In that respect tumor spheroids have established themselves to be one of the most common and functional scaffold free technique for 3D cell culture. These models are considered to be self-assembling or grow as cell clusters beginning from single-cell suspensions (Zhao et al. 2012). Spheroids obtained from numerous cell types have been successfully used to investigate reactions to different treatment methods which includes immunotherapy, chemotherapy, radiation, or a combination of various therapeutics. Spheroids formulated from primary tumors have been shown to modulate cell–cell interaction, communication channels such as gap junctions, desmosomes, etc. which provide us with information related to the molecular mechanisms controlling cellular proliferation and differentiation in tumors (Mueller-Klieser 1997). Ehsan et al. constructed a pre-vascularized tumor model by incorporating spheroids of endothelial cells and either cancer cells including MCF-10A, MDA-MB-231, A549, SW620 in fibroblast containing fibrin matrix. The endothelial cells produced a significantly high number of sprouts and also vascularized the spheroids with blood vessels of irregular morphology (Ehsan et al. 2014). Ghosh et al., developed an in vitro melanoma angiogenesis model employing multicellular tumor spheroids arising from differentiated (HBL) or undifferentiated (NA8) melanoma cell lines with the aim to determine the correlation of expression with respect to the differentiation marker of cancer cells along with angiogenesis (Ghosh et al. 2007). We screened 15 various melanoma cell lines to determine the expression of Melan-A/MART-1, gp100, and tyrosinase differentiation antigens. Although HBL cell line showed differentiation, however, NA8 cell line exhibited de-differentiation. The NA8 spheroids showed the upregulation of the genes encoding VEGF, Ephrin A1 and Angiopoietin-like 4 in comparison to that in the monolayer. Contrastingly, there was absence of these genes in HBL cultures except the ANGPTL4. When the Na8 cells were cocultured with HMEC, an enhanced endothelial cell networking was observed in NA8 spheroid. To our surprise, HMEC cells were repelled by the HBL cells presumably as a result of semaphorin 6D. It is interesting to note that the overexpression of T-cadherin in HMEC cell line gave rise to enhanced network formation in NA8 spheroids, however, cells could not overcome the repulsive effects of the differentiated HBL spheroids. This elegant in vitro model also elucidated how a cluster of cancer cells might cause perforations in blood vessels, and initiate metastasis. (Ghosh et al. 2007).
5. Role of tumor organoid models in understanding angiogenesis mechanisms
  Organoid models, which are in vitro 3D tissue constructs derived from either stem cells or iPSCs mimicking the in vivo environment of the corresponding organ holds promising implications in studying human development and diseases. The 3D spatial arrangement of organoid is due to its ability to self-organize by cell sorting and in a spatially confined lineage commitment (Takebe et al. 2017). Wörsdörfer et al*.,* generated complex tumor organoids by employing mesodermal progenitor cells (MPCs) to overcome the limitations of iPSCs-derived organoids including lack of stroma, immune cells, and a functional vasculature. The model thus developed, demonstrated typical features of a blood vessel including, endothelial cell junctions, luminal caveolae, basement membrane, and macrovesicles (Wörsdörfer et al. 2019). In recent years, organoid models have been extensively explored to study various aspects of tumor biology including angiogenic signaling pathways, screening drugs, and construct biobanks. For example, Wang et al. using organoids of hepatocellular carcinoma cells cultured with endothelial cells and fibroblasts revealed the expression of EMT markers including vimentin, TGF-β, and MMP9, neoangiogenesis markers such as VEGFR2, HIF-1α, and VEGF, inflammatory markers such as CXCL12, CXCR4, and TNFα to be upregulated in these organoids (Wang et al. 2017). Shirure et al*.* constructed PDMS-based tumor organoids derived from colorectal cancer cell lines Caco-2 and CRC-268, breast cancer cell line MDA-MB-231, and MCF-7, and Patient-derived organoids (PDOs) derived from breast cancer patients which consisted of immune cells, ECs, fibroblasts, and cancer cells. These tumor PDOs when cultured adjacent to the vascular network showed profound angiogenic response for as long as 22 days. Culturing these tumor organoids adjacent to cancer-associated fibroblasts (CAF) showed a statistically significant increase in the expression of VEGF-A and TGF-β relative to normal fibroblasts revealing the genomic diversity of CAFs (Shirure et al. 2018). Mazio et al. constructed vascularized 3D breast tumor models consisting of HUVEC, MCF-7, and human fibroblasts embedded in an ECM rich in collagen and hyaluronic acid. Statistically significant “capillary-like structures” with enhanced diameter and branching were observed in MCF-7 embedded tumor models compared to control. The authors also reported the formation of vessel-like structures consisting of tumor cells parallel to capillary-like structures which later merged to mosaic vessels (Mazio et al. 2018). Vlachogiannis et al*.,* used PDO models to establish a living biobank of 71 patients with gastrointestinal cancers providing valuable insights into histopathological markers of colorectal cancer including CDX-2 and CK7. Next-generation sequencing of these PDOs showed 96% overlap in gene expression with their parental biopsies with mutations in key genes such as APC, KRAS, TP53, MYC, and EGFR. These PDOs on treatment with regorafenib, an anti-angiogenic drug, showed a marked reduction in tumor angiogenesis (Vlachogiannis et al. 2018; Khan et al. 2018). Sobrino et al*.* constructed PDMS-based vascularized micro tumors by co-culturing one of the tumor cells- HCT116, SW620, SW480, MCF-7, MDA-MB-231, and MNT-1 with endothelial colony-forming cells derived endothelial cells and human lung fibroblasts. These models when treated with different anti-cancer drugs including sorafenib showed a marked reduction in tumor growth, and angiogenesis (Sobrino et al. 2016). Bayat et al. engineered a HUVEC vascularized 3D tumor model of glioblastoma to test the efficacy of the drug atorvastatin. The authors found that atorvastatin downregulated the expression of endothelial markers such as CD31, VEGF, and Bcl-2 and induced the expression of caspase-3 revealing its anti-angiogenic and apoptotic effect (Bayat et al. 2018).
  Organoid models have provided incredible scope for tumor modeling and pathogenesis, screening anti-cancer drugs, and personalized medicine. Tumor models are also developed by editing genomes using the Clustered regularly interspaced short palindromic repeats-CRISPR-associated protein 9 (CRISPR/Cas9) technique for studying the genetic basis of diseases. Freedman et al. demonstrated the construction of genetically modified kidney organoids to study genetic kidney disease (Freedman et al. 2015). Organoids were used to determine the optimized treatment strategy for individual patients. Living biobanks of patient-derived organoids are being developed. Organoid biobanking involves the collection of tissue resections after surgery and developing them into organoids that can be used immediately as live culture or frozen in liquid nitrogen (van de Wetering et al. 2015).
  The major challenge in developing a tissue engineering-based organoid system is that after reaching a particular size, cells stop proliferating and acquire a core of necrotic cells. The major reason for the formation of the necrotic core is the wall thickness of the tissue-engineered grafts since wall thickness greater than 150 µm limits the diffusion of nutrients and gases. However, to enhance graft survival rates, the graft should be pre-vascularized to allow proper inosculation with the vascular network of the host (Sharma et al. 2019). To overcome this limitation, several approaches in microfluidics have been developed. For example, during the process of fabrication, micro-molds, sacrificial materials, liquid templates provide support to geometries inside hydrogel materials (McNulty et al. 2019). Hydrogel-based perfusable vascularized tissues help in modeling the in vitro processes better and enhances the efficiency of in vivo transplantation. Another approach, stereolithography, which involves the cross-linking of photosensitive polymers using ultraviolet light can be used to create perfusable channels by embedding a sacrificial filament network on a matrix consisting of cells. These channels can then be implanted with endothelial cells for the formation of a functional vascular system (Miller et al. 2012; Grebenyuk and Ranga 2019). Hydrogels, soft polymer materials containing 3D structural networks, are preferred for the construction of vascular tissues due to their ability to retain water in 3D networks. As hydrogels consist of interconnected pores filled with water, they enable easy diffusion of nutrient molecules, which is the primary function of a blood vessel. Natural hydrogels such as matrigel, collagen, fibrin, and gelatin have been extensively used in vascular tissue engineering. Matrigel is a soluble extract from extracellular matrix proteins derived from mice tumors that polymerizes at 24 °C–37 °C to form a 3D gel (Kleinman and Martin 2005). Collagen gels are the result of interactions and assembly of collagen I fibrils while fibrin is produced by thrombin’s cleaving action on fibrinogen (Li et al. 2015). Some of the protein-based hydrogels, polysaccharides like chitosan (produced from chitin), and alginate have also been explored. Crosslinking of alginate molecules are much faster compared to other biomaterials and hence are widely used to produce 3D matrix in tissue engineering. Hydrogels are formed under different conditions by modifying their constituent materials such as polyethylene glycol, polyvinyl alcohol, polyhydroxy ethyl methacrylate, and polyacrylamide based on their cellular compatibility. To fabricate the engineered tissues, cells should be enveloped into the hydrogel. Additive manufacturing (layer by layer deposition), selective removal of materials for the formation of tubular voids connected to perfusion networks, and use of sacrificial materials are some of the 3D fabrication techniques explored (Grebenyuk and Ranga 2019; Xie et al. 2020). Further, 3D bioprinting technique can be of significance in developing pre-vascularized grafts. As mentioned earlier, recapitulation of the embryonic level vascular developmental pathways by 3D bioprinting might help to enhanced pre-vascularization in the tissue-engineered models (Sharma et al. 2019).
6. Organ on chips—Bridging the gap between 2D and 3D models
  Organ on chips (OOC) is micro-engineered biomimetic systems that reciprocate the complex structure, microenvironment, and functionality of parent organs such as liver, heart, lung, kidney, brain, bone, and also vasculature. OOC system facilitates the direct, real-time visualization of complex and integrated organ-level responses to various biomechanical and biochemical stimuli unlike the conventional cell culture models (Esch et al. 2015). These models bridge the gap between the conventional 2D, 3D cell cultures and in vivo models. A single cell, tissue, or many connected models are arranged in a microfluidic flow device (Haddrick and Simpson 2019). These devices allow the investigators to study the cellular, molecular, biochemical, and biophysical characteristics in a precisely constrained manner (Sontheimer-Phelps et al. 2019). For example, Kim et al. constructed a PDMS-based perfusable microvascular network in fibrin bioink consisting of heterogenous cell types including HUVEC, lung fibroblasts, pericytes, and glioblastoma cell line U87MG. The authors demonstrated the establishment of vasculature with aberrant morphology, multiple tip cell formation, poorly perfusable immature vessels in response to cancer cell secretome (Kim et al. 2013). Similarly, Chung et al. using PDMS-based microfluidic scaffold coated with a monolayer of HMVEC demonstrated significant growth of angiogenic sprouts in response to rat mammary adenocarcinoma cell line MTLn3 and human glioblastoma cell line U87MG. Interestingly, the authors found HMVEC exhibited a greater migratory response to MTLn3 cells than U87MG (Chung et al. 2009). Buchanan et al*.,* using a 3D microfluidic breast tumor vascular model of MDA-MB-231 which showed that endothelial cells displayed enhanced permeability and tumor cells downregulated their expression of angiogenic genes such as MMP9, HIF-1, VEGF-A, ANG1 and ANG2 in response to high microvascular wall shear stress of 10dyn/cm^2^ revealing the effect of mechanical forces in tumor angiogenesis (Buchanan et al. 2014). Studies have shown tumors to regulate the velocity of fluids flowing through their vasculature to be low thereby upregulating the expression of angiogenic genes (Walker-Samuel et al. 2018). Lee et al*.,* developed a perfusable PDMS-based 3D microfluidic model consisting of HUVEC, lung fibroblasts, and U87MG embedded in fibrin gel. A significant reduction in the sprout length, the number was seen when these models were treated with bevacizumab, an anti-angiogenic drug (Lee et al. 2014). Another microfluidic model employing MDA-MB-231, HUVEC, macrovascular endothelial cells, and macrophages in an ECM matrix to demonstrate the role of macrophages in the process of intravasation was developed by Zervantonakis et al. The authors observed a ninefold increase in tumor cells undergoing intravasation when compared to control, guided by macrophage-derived TNF-α induced permeability of the endothelial barrier. This model highlighted the need for the incorporation of immune cells in 3D models focusing on constructing translational models mimicking tumor microenvironment (Zervantonakis et al. 2012).
  Vasculature in solid tumors is highly dysregulated as it is influenced by numerous angiocrine molecules present in the heterotypic secretome of the tumor niche (da Cunha et al. 2019). The complex molecular interplay between the tumor cells and non-tumor cells such as fibroblasts, adipocytes, monocytes, macrophages, endothelial cells, and cancer stem cells leads to the synthesis of these angiocrine molecules and result in the development of blood vessels with aberrant morphology and functionality. 3D models employing all these heterogenous cell types would better simulate the autocrine, paracrine, and juxtacrine signaling pathways regulating tumor angiogenesis. Further, tumor ECM is structurally and biochemically distinct compared to the ECM of normal tissue. The tumor ECM comprises of diverse components including proteoglycans such as syndecan, glypican, versican, aggrecan; polysaccharides such as hyaluronic acid; proteins such as collagen, fibulin, laminin, fibrillin, fibronectin, periostin, thrombospondin, and integrin (Nallanthighal et al. 2019). Various studies have shown ECM to modulate tumor angiogenesis by (a) regulating the secretion of pro-angiogenic factors, (b) angiogenic tip cell formation, (c) providing physical space for vessel formation (Campbell et al. 2010; Mongiat et al. 2016). Hence, integrating key ECM components such as collagen, integrin, syndecan, glypican, and perlecan which have been shown to regulate tumor angiogenesis would replicate the in vivo tumor vasculature to a greater extent. Emerging evidences have surfaced the role of the nervous system in regulating tumor angiogenesis (Jiang et al. 2020). For example, catecholamines including epinephrine and norepinephrine have been shown to induce the expression of pro-angiogenic factors such as VEGF, MMP2, MMP7, MMP9, and IL-8 in tumor resident cells (Chen et al.; Shi et al. 2013). In contrast, dopamine has been shown to inhibit the expression of VEGF and MMP9 (Chakroborty et al. 2009). Several other neurotransmitters such as gamma amino butyric acid, 5-hydroxytryptamine, acetylcholine, neuropeptide Y, and nitric oxide have been shown to influence the development of tumor vasculature in various cancer types including breast cancer, colorectal cancer, melanoma, lung cancer, ovarian cancer, gastric cancer, and neuroblastomas (Kuol et al. 2018). Hence, developing 3D multicellular tumor vascular models consisting of neurons would unravel the role of these neurotransmitters in the tumoral arena. Accumulating evidence from next-generation sequencing have revealed patient-specific and tissue-specific genetic and epigenetic alterations in the tumor resident cells (Gerlinger et al. 2012; Wragg and Bicknell 2015; Zhang et al. 2018). However, most of the existing 3D models employ established cell lines that may fail to simulate the in vivo tumoral response that have urged the development of 3D models employing parental tissue-derived cells. However, failure to recapitulate the systemic changes, lack of endocrine signaling, use of cell lines instead of primary cells have curtailed their progress into translational research.
  Many studies have attempted to mimic the angiogenic tumor microenvironment using microfluidic devices. One such device was used to show the role of phthalimide compounds in reducing angiogenesis by observing the sprouting ability of endothelial cells after treatment (Mercurio et al. 2019). Treatment of tumor organoids with tyrosine kinase inhibitor sorafenib showed a disturbance in the formation of endothelial networks. Thus, this model allowed examining the responses of anti-angiogenic compounds as well as drug testing applications. The co-culturing of mesenchymal progenitor cells and neural organoids generated complex vascularized neural organoids. This showed the mesenchymal/epithelial interactions induced the perineural vascular plexus formation. The maturation of pre-formed organoid vessels requires the perfusion of the vascular network. Pre-vascularized organoids produced in vitro, when transplanted on the chicken chorioallantoic membrane, connected to host circulation (Wörsdörfer et al. 2019).
7. Challenges in developing appropriate vascular 3D model systems
  Albeit, diseases related to aberrant vasculature are broadly classified into excessive and insufficient angiogenesis, paracrine interactions, and the key mediators to modulate pathological angiogenesis are highly heterogeneous and thus specific to diseases. For example, in the tumor microenvironment, the interaction of tumor cells, stromal cells, and infiltrating immune cells along with endothelial cells of abnormally enhanced cytokines and growth factors secretions are responsible for leaky and tortuous blood vessels. On the other hand, intra-plaque angiogenesis in atherosclerosis plaques is regulated by interaction between foam cells, smooth muscle cells, and endothelial cells. This suggests, an absolute requirement for developing disease-specific vascular models.

**Table 1:** In vitro, In vivo, and Ex vivo models of angiogenesis

	Assays	Technique	Scientific readout	Advantages	Disadvantages
In vitro	Endothelial cell proliferation assay	Cell counting	Percentage of viable cells	Quantification of proliferating cells, apoptotic cells, and DNA content of the cell	Prone to manual error
		MTT assay	Measuring succinate dehydrogenase activity	Less expensive	Time-consuming
		3H-Thymidine /Bromouridine incorporation assay	DNA/RNA synthesis		
	Endothelial cell migration assay	Boyden chamber	Cell migration and invasion	Determination of migratory capacity of cells	Technical difficulties
		Matrix degradation assay	Molecular factors influencing migration	Quantification of the rate of migration	Time-consuming
		Wound healing	Directional cell migration		Low rate of reproducibility
					Unable to accurately determine differences between proliferation and migration
	Endothelial cell differentiation assay	Matrigel assay	Rearrangement of cells to form tubules	Quantification of pro-angiogenic factors	Technical difficulty
		3D spheroid assays	Paracrine interactions and modulated pathways	Influence of biomolecules on ECs	Time-consuming
		Co-culturing ECs with other cell types			
In vivo	Matrigel plug assay	Immuno-histochemistry staining	Quantification of newly formed blood vessels	Ideal model to study tissue regeneration	Expensive
					Time-consuming
	CAM assay	Immuno-histochemistry staining	Formation of new blood vessels	Evaluation of angiogenic response	Sensitivity of the membrane to oxygen tension
	Corneal angiogenesis assay	Microscopic observation	Vessel length and vascular sprouts	The reliable method as the cornea is devoid of pre-existing vasculature	Inappropriate for large scale studies
		Immuno-histochemistry staining			
	Rodent mesentery angiogenesis assay	Immuno-histochemistry staining	Percentage of vascularized area	Extremely thin tissue enables easy visualization	Difficulty in quantification of angiogenesis
				High sensitivity	
Ex vivo	Rat aortic ring assay	Microscopic observation	Angiogenic sprouts and vessel length	Mimics in vivo conditions	Vessel growth is influenced by surrounding tissue
	Chick aortic arch assay	Microscopic observation	Cellular proliferation, migration, tube formation and vessel branching	Less expensive and less experimental time	Vessel growth is influenced by surrounding tissue
	Rodent ear angiogenesis assay	Intravascular staining with biotinylated lectin	Vessel growth and branching	Easy visualization	Vessel growth is influenced by host cell interactions
				Mimics in vivo conditions	
	Mouse fetal metatarsal angiogenesis assay	Immuno-histochemistry	Vessel sprouts, molecules influencing angiogenesis	Better representative of in vivo sprouts	Devoid of biomechanical force influencing the phenotype
				Employs microvascular cells	Requires technical precision

**Table 2:** Tissue engineering-based pre-vascularization strategies

Mechanical stimulation				
Biomaterial	Cell type	Strategy	Application	Reference
Collagen hydrogel	Rat microvessel fragments	Static external loader cyclic external load	Angiogenic microvessel	Krishnan et al. (2008)
Fibrin gel	Human blood outgrowth endothelial cells	Cell-induced gel compaction	Aligned microvessels	Morin et al. (2013); Herrera-Vizcaíno et al. (2019)
Electrical stimulation				
Biomaterial	Cell type	Strategy	Application	Reference
Matrigel	Human mammary epithelial cells, HUVECs	DC electric field	Directional migration of cells	Bai et al. (2004)
Surface topography				
Biomaterial	Cell type	Strategy	Application	Reference
Silk fibroin and fibrin	HUVECs + human foreskin fibroblasts	3D porous scaffolds	Capillary‐like structure formation	Samal et al. (2015)
Silk fibroin fibers in poly (d,l-lactic acid) porous scaffolds	Human endothelial cells	3D salt-leached scaffolds	In vitro endothelial and to promote vascularization in vivo	Stoppato et al. (2013)
Gelatin methacrylate hydrogels	Human blood-derived endothelial colony-forming cells and bone marrow-derived mesenchymal stem cells	3D porous scaffolds	The functional human vascular network	Chen et al. (2012)
Silk fibroin	Human microvascular endothelial and osteoblast cells	3D fibrous scaffolds	Anastomosis of neo-microcapillaries with the host vasculature	Unger et al. 92010)
Collagen	Endothelial colony-forming cells and endothelial progenitor cells	3D fibrous scaffolds (varied collagen concentration)	Guiding in vivo vascularization	Critser et al. (2010)
Decellularized fibroblasts derived ECM	Human mesenchymal stem cells	3D nanofibrous scaffolds	Engineering organized tissues	Xing et al. (2014)

Further, recent advancements in vascular biology have revealed the existence of a structurally and functionally heterogeneous population of endothelial cells along the vascular tree (Aird 2012). Mounting pieces of evidence indicate intricate cellular and molecular interactions between the constituent cells of the blood vessel during angiogenesis. Also, various factors such as shear stress and interactions of blood constituents with the cells of the vessel significantly influence the vascular tone and functionality of the surrounding ECs. Understanding these underlying mechanisms might help us formulate therapeutic strategies against various vascular disorders including stroke, myocardial infarction, wound healing, diabetic vasculopathy, rheumatoid arthritis, and solid tumors. Although several 3D model systems consisting of ECs alone have been successfully developed, their in vivo implantation has resulted in immature integration to the host vasculature leading to regression (Au et al. 2009). Multicellular organoids developed by 3D fabrication of heterogeneous population of cells derived from stem cells or iPSCs (ECs, pericytes, and smooth muscle cells) derived from the same individual would possibly integrate better with the host vasculature and re-vascularize the damaged tissue. Nevertheless, once the mature, dense, and organized microvascular tissue/organ graft is obtained it is quintessential to optimize, and limit the volume of the perfusable fluids that can be perfused through these grafts without affecting the structural and mechanical integrity.

Studies have demonstrated the existence of a highly complex tumor microenvironment due to various pro-angiogenic molecules secreted by numerous constituent cell types. The development of multicellular organoids consisting of tumor-associated cells such as tumor-associated-adipocytes, fibroblasts, and macrophages would better mimic the cytokine storm of the tumor, helping us gain deeper insights into its cellular and molecular consequences. Intra-individual variations in response to anti-angiogenic drugs due to differential gene expression of endothelial cells among the population often pose an obstacle to for clinical management of solid tumors. Such variations might be managed by prescribing patient-specific anti-angiogenic drug(s) profiles generated by in vitro screening of available drugs on multicellular vascular organoids developed by patient-derived cells. Endothelial cells are quiescent in a normal physiological state and survive with minimal metabolic needs. However, in pathological conditions, studies have shown reprogramming of endothelial cell metabolism due to (epi)genetic changes and also as a consequence of continuous interactions with other cell types. To fulfill the energy demands of the cell, rapidly proliferating tumor ECs are known to exist in a hyper glycolytic state by overexpressing phosphofructo-2-kinase/fructose 2,6 bis-phosphatase 3, the glycolytic regulatory enzyme (Cantelmo et al. 2016). Proliferating ECs have been shown to not only upregulate fatty acid synthase but also upregulate the expression of fatty acid transport protein-3 (FATP) and FATP-4 (VEGF-B signaling axis), thereby feeding fatty acids (FAs) to the anabolic pathways of signaling molecules, phospholipids for cell membrane synthesis, and FA oxidation (Hagberg et al. 2010). Tumor endothelial cells are also known to overexpress phosphoglycerate dehydrogenase, the regulatory enzyme of the de novo biosynthetic pathway of serine, cysteine, and glycine which is involved in nucleotide biosynthesis (Falkenberg et al. 2019). This indicates a successful vascular organoid model which also requires tailoring metabolic needs and accordingly defined nutrient supply for its longer sustenance and anastomosis.

In the context of tissue engineering, advanced 3D bioprinting techniques should aim towards the fabrication of physiological scale vascular tissues/organs while taking care of the recapitulation of microvascular features. Moreover, 3D bioprinting-based tissue engineering technique could be successfully modulated for developing vascular constructs mimicking the native ECM thus helping to avoid the issues related to cell homing, which is a major issue observed in cell therapy based early phase clinical trial (Deveza et al. 2012). 3D bioprinting-based tissue engineering approaches have been used to modulate the supply of exogenously provided growth factors to regulate the growth, perfusion, and anastomosis of the neovascular grafts. Strategically designed bioinks would play a crucial role in such a scenario since the required growth factor can be immobilized onto the bioink to allow their slow and sustained release in a spatially and temporally controlled manner during in vitro culture and post-implantation.

## Conclusion

Cellular and molecular signaling during tumor angiogenesis are diverse in the context of (a) paracrine and autocrine interactions, (b) magnitude, and (c) spatial and temporal expression of key angiogenic modulators compared to that of other tissues and pathologies. This indicates requirement of developing vascular models specific to tumor tissues to understand intricate processes of tumor angiogenesis and clinical applicability. In recent years, organoid technology involving with the ability to maintain the cellular heterogeneity to mimic in vivo tumor microenvironment and to deposit cells beyond the diffusion limit of oxygen, a delimitation of conventional models, has given new dimensions to in vitro cell culture models. Vascularized tumor organoid models involving multiple cell types also enables to understand complex interactions in tumor microenvironment.

## Data Availability

Not applicable.

## Abbreviations

2D: Two dimensional
3D: Three dimensional
ANGPTL4: Angiopoietin-like 4
CAF: Cancer-associated fibroblast
CAM: Chorioallantoic membrane
CD: Cluster of differentiation
DNA: Deoxyribose nucleic acid
EC: Endothelial cell
EPC: Endothelial progenitor cell
ECM: Extracellular matrix
eNOS: Endothelial nitric oxide synthase
EGF: Epidermal growth factor
EMT: Endothelial mesenchymal transition
FA: Fatty acid
FATP: Fatty acid transport protein
FGF: Fibroblast growth factor
GFP: Green fluorescent protein
HDMEC: Human dermal microvascular endothelial cell
HGF: Hepatocyte growth factor
HIF: Hypoxia-inducible factor
HMVEC: Human microvascular endothelial cell
HUVEC: Human umbilical vein endothelial cell
IFN: Interferon
IL: Interleukin
iPSC: Induced pluripotent stem cell
MMP: Matrix metalloproteinase
MPC: Mesodermal progenitor cell
OOC: Organ on chip
PDGF: Platelet-derived growth factor
PDMS: Poly dimethyl siloxane
PDO: Patient-derived organoid
PEG: Poly ethylene glycol
Poly-HEMA: Poly hydroxy ethyl methyl acrylate
RFP: Red fluorescent protein
SDF: Stromal derived growth factor
SF–G: Silk fibroin–gelatin
SMC: Smooth muscle cell
TGF-β: Transforming growth factor-β
TNF-α: Tumor necrosis factor-α
TIMP: Tissue inhibitor of metalloproteinase
VEGF: Vascular endothelial growth factor
VEGFR: Vascular endothelial growth factor receptor
VM: Vasculogenic mimicry
vWF: Von Willebrand factor
